# Supplementary material for: Impact of face masks and sunglasses on emotion recognition in South Koreans
Source: PLoS One. 2022 Feb 3;17(2):e0263466. doi: 10.1371/journal.pone.0263466 (PMC8812856; doi:10.1371/journal.pone.0263466)
Supplement: Attachment — Submitted filename: Rebuttal Letter.docx [file pone.0263466.s001.docx]

**Rebuttal Letter**

Dear Managing Editor,

PLOS ONE Reviews

Thank you for inviting us to submit a revised draft of our manuscript entitled, “Does masking one's facial area masks emotions? The impact of face masks and sunglasses on emotion recognition in South Koreans (revision)” to PLOS ONE. We express our cordial gratitude for the valuable and constructive review of our paper. The reviewers’ comments have helped us to further strengthen the overall quality of the paper. We have incorporated changes that reflect the detailed suggestions you have graciously provided. We also hope that our edits and the responses we provide below satisfactorily address all the issues and concerns you and the reviewer have noted. The specific responses to the reviewer’s comments are listed below.

**The Editor’s comments:**

**Comment 1:** You have update your Introduction and Discussion taking into account latest studies in the field.

**Response:**

Thank you for the suggestion and recommending the recent papers on this topic. We have updated the introduction in the following manner:

We cited the paper by Pavlova and Sokolov (2021) and introduced the most recent relevant studies that were reviewed in the paper. Specifically, we incorporated the findings from Carbon & Serrano (2021) and Noyes et al., (2021) which were the most up-to-date studies that tested the effects of masked faces on emotion recognition. In addition, following reviewer 1’s suggestion, we have significantly reduced the part on the findings of Ruba & Pollack’s (2020) study. Furthermore, we updated the findings that gender differences exist in facial expression recognition of covered faces with the latest study synthesizing face masks.

In the discussion section, we provided a more comprehensive discussion by comparing our findings with Noyes et al. (2021) as well as Carbon (2020). In addition, we updated the latest studies in regard to reading emotions among children.

**Comment 2:** the title should be modified to better reflect the specificity and novelty of your study.

**Response:** As per your suggestion, we have changed the title to “Does masking one's facial area masks emotions? The impact of face masks and sunglasses on emotion recognition in South Koreans”. The manuscript title has been updated and now it is more meaningful.

**Comment 3:** did you check the data sets for normality of distribution and how? Please make a clear statement on it in the Method Section.

**Response:** Thank you for your suggestion. We tested normality of distribution and found that some variables were not normally distributed. However, we decided to continue using ANOVA as of the robustness of the statistic against violations of normality with equal sample sizes (Glass et al., 1972). The ANOVA is reasonably robust to violations of homogeneity of variance when group sizes are equal, and minor violations of the normal distribution can also still produce results that are similar to those when the data are normally distributed (Schmider et al. 2010). We have confirmed that many studies use the repeated measures ANOVA based on F test's robust to non-normality, even if the data are nonnormal (Ackermann et al., 2019; Adriaens et al., 2018; Everman et al., 2018; Ghazali et al., 2018; Goheen et al., 2013; Savage et al., 2020; Zhang et al., 2018). We also described that the violation of normality assumption and the reason we still use within-subject ANOVA in the Results section (p. 9, lines 184-187). Nevertheless, if you still think to need a nonnormality test, we will proceed with a new analysis.

“Before analyzing the main results, all data were checked for normality distribution. Normality was violated for some variables but we decided to use the repeated measures ANOVA because ANOVAs are generally considered robust to nonnormality with sample sizes being equal (within-subject design) [34].”

Additionally, we added a sentence related to how to present results according to the tests of sphericity (p. 9, lines 189-190).

“We reported the results of Greenhouse–Geisser correction whenever Mauchly’s test of sphericity was significant.”

References

Glass G V, Peckham PD, & Sanders JR. Consequences of failure to meet assumptions underlying the fixed effects analyses of variance and covariance. Review of educational research. 1972;42(3): 237-288.

Schmider E, Ziegler M, Danay E, Beyer L, Bühner M. Is it really robust?. Methodology. 2010 Sep 8.

Ackermann C, Beggiato M, Bluhm LF, Löw A, Krems JF. Deceleration parameters and their applicability as informal communication signal between pedestrians and automated vehicles. Transportation research part F: traffic psychology and behaviour. 2019;62: 757-768.

Adriaens K, Van Gucht D, Baeyens F. IQOSTM vs. e-cigarette vs. tobacco cigarette: a direct comparison of short-term effects after overnight-abstinence. International journal of environmental research and public health. 2018;15(12): 2902.

Everman ER, Delzeit JL, Hunter FK, Gleason JM,Morgan TJ. Costs of cold acclimation on survival and reproductive behavior in Drosophila melanogaster. PLOS ONE. 2018;13(5): e0197822.

Ghazali AS, Ham J, Barakova E, Markopoulos P. The influence of social cues in persuasive social robots on psychological reactance and compliance. Computers in Human Behavior. 2018;87: 58-65.

Goheen JR, Palmer TM, Charles GK, Helgen KM, Kinyua SN, Maclean JE, ... Pringle RM. Piecewise disassembly of a large-herbivore community across a rainfall gradient: the UHURU experiment. PLOS ONE. 2013; 8(2): e55192.

Savage MJ, James R, Magistro D, Donaldson J, Healy LC, Nevill M, Hennis PJ. Mental health and movement behaviour during the COVID-19 pandemic in UK university students: Prospective cohort study. Mental Health and Physical Activity. 2020;19: 100357.

Zhang Z, Zhang B, Cao C, Chen W. The effects of using an active workstation on executive function in Chinese college students. PLOS ONE. 2018;13(6): e0197740.

**Comment 4:** Did you analyze the effect of observers' gender on performance?

**Response:** Yes. We described this at the beginning of the Results section. However to express it more effectively, we have revised the text to “However, the main effect of the sex of the participants and the interaction between the sex of the participants and the sex of the face stimuli were not statistically significant, F(1,37) = 1.74, p = .20; F(1, 37) = 1.42, p = .24; respectively.” (p. 17, lines 338-341).

**The Reviewer's comments:**

**Comment 1:** The authors claim that the Ruba & Pollack study was “particularly informative”; I would like to disagree, and the authors sum this up competently later on: That study was a (very) limited one; additional to the critical points the authors already raised, I would like to add that the material and experimental design was highly problematic as we cannot assign the variance to the specific face or the specific intervention (type of covering) as different faces were used across conditions—in the end, the authors devote a very long section on that paper but the validity of it is currently debated. Please consider new studies which addressed these critical points, e.g. Carbon & Serrano (2021).

**Response:** Thank you for pointing this out. The editor also expressed the same concern. Based on your and the editor’s recommendation, we have updated the introduction in the following manner:

We cited the paper by Pavlova and Sokolov (2021) and introduced the most recent relevant studies that were reviewed in the paper. Specifically, we incorporated the findings from Carbon & Serrano (2021) and Noyes et al., (2021) which were the most up-to-date studies that tested the effects of masked faces on emotion recognition. In addition, following reviewer 1’s suggestion, we have significantly reduced the part on the findings of Ruba & Pollack’s (2020) study. Furthermore, we updated the findings that gender differences exist in facial expression recognition of covered faces with the latest study synthesizing face masks.

In the discussion section, we provided a more comprehensive discussion by comparing our findings with Noyes et al. (2021) as well as Carbon (2020). In addition, we updated the latest studies in regard to reading emotions among children.

**Comment 2:** G_Power -analyses: this makes sense to conduct such an analysis before conducting the study, but why then were 39 instead of 28 persons tested. Was the test power analysis in the end just done for the sake of having done it? Please verify this.

**Response:** First, we calculated the number of participants required in order to meet the assumptions of independence and normality in our experiment using G-power. After that, we recruited more participants than we needed in anticipation of dropouts during the experiment or malfunctioning experimental computers or programs. In addition, more participants were recruited for more stable results because there may be participants who did not meet the pre-set exclusion criteria (less than a 50% correct facial expression recognition answer rate when presented with a fully visible face without a face mask or sunglasses were excluded). A total of 40 participants were recruited, but all 39 were used in the analysis because there was no reason to exclude them from the data except one. (p. 6, lines 130-134).

“However, we sampled more participants than the required number for potential participants who would get excluded if computers malfunction or those who did not meet the pre-set exclusion criteria (less than a 50% correct facial expression recognition answer rate when presented with a fully visible face without a face mask or sunglasses were excluded). This pre-determined criterion was based on that of Carbon’s method [9].”

**Comment 3:** Title : this is typically NOT a major issue, but here the title is so underspecified that I would not recommend.

**Response:** As per your suggestion, we have changed the title to “Does masking one's facial area masks emotions? The impact of face masks and sunglasses on emotion recognition in South Koreans”.

**Comment 4:** Please reduce the “precision” with values which are imprecisely measured, e.g. age.

**Response:** We did not understand this comment. Could you please explain what you meant by “reduce the precision”? Thank you.

**Comment 5:** Please add units to values (e.g., again, age).

**Response:** We have reflected this comment by adding units to age (p. 2, lines 23; p. 7, line 143).

“mean age = 24.23 years”

*“M_age_* = 24.23 years, *SD_age_* = 4.68 years”

**Comment 6:** Greek letters should not be italicized.

**Response:** Thank you for your comments. We have revised the reporting of all partial eta-squared (ηp2) statistics throughout the paper. You can check them without italics.

**Comment 7:** It is great that you have offered the data on OSF! Please consider (later on) to provide the analyses, too

**Response:** Thank you for your suggestion. We will update with the syntax for the data analyses before the paper is published.

**Comment 8:** Figure 1 is very illustrative, thank you! I would extend the arrow and put a “…” at the end, juts to make clear it goes on and on and that a trial is NOT a couple of two pictures but that this diagram just shows a sequence of two typical trials (the quality of the scale could be increased in the final version—it is hard to read currently).

**Response:** We have now figure 1 with arrows and “…”. We think these changes now better to describe our trials. We hope that you agree.

**Comment 9:** Please insert spaces between values and units

**Response:** We checked it throughout the paper.

**Comment 10:** Please add effect sizes, also to the Chi2-tests.

**Response:** We calculated effect size of the χ^2^ using Cramer's V which is for categorical comparisons. Cramer's V coefficient and p-value were reported (p. 9, lines 180).

“($\chi^{2}$ = 21312.458, *p* < .001, Cramer’s V = 0.601, *p* < .001)”

**Comment 11:** Fig.2 is very nice, but please add the method how you analysed the pairwise comparisons; was it alpha-corrected? How were the error bars calculated? What do they represent? Just make everything clear, please!

**Response:** We computed a repeated-measures ANOVA and pairwise comparisons using SPSS 25 software, and constructed Fig 2 using the alpha and standard errors provided by the package. We agree with you and have clarified how we analyzed (under the Fig.2). We think this change now is better for readers to understand our analysis. We hope that you agree.

**Comment 12:** “subject”: I would (just a recommendation, no rule or obligation—personal taste) not speak of “subjects” but persons / participants / etc.

**Response:** We have reflected this comment by replacing the term “subject” throughout the paper with “participants”.

**Comment 13:** Discussion: About video material : please check the literature as there are already papers on video-related mask research.

**Response:** Thank you for the reminder. As you have mentioned about existing literature on video materials, we managed to find that a paper with regards to video materials with faces wearing masks and thus, included it in our Discussion section. However, since the paper has limitations in that, it contains only two emotions, happy and sad, we decided to maintain the argument that video research is necessary for the effect of masks on facial expressions recognition (p. 20, lines 418-422; 424-426).

“Recently, researchers measured the accuracy of emotional recognition using video stimulation, which adds a static background to the dynamic facial expression set [40]. However, since this study included only two emotions, happiness and sadness, and sad, it is difficult to grasp the effect of on recognition of various emotions in facial expression.”

“Thus, future studies using video stimuli that include various emotions and involve other sources of information would render a more ecologically valid effect of facial occlusion on emotion recognition.”

**Comment 14:** Baron-Cohen’s study [see ref.1] was additionally tested by Schmidtmann and colleagues with non-clinical participants meanwhile. Please check.

**Response:** We checked the study of Schmidtmann et al. (2020) and changed the references. Thank you for your suggestion.

**Comment 15:** Discussion: children : see the latest paper on children-related samples

**Response:** We checked the paper (Carbon & Serrano, 2021) that examined the effect of masks on facial expression recognition in children and added it in the corresponding part. (p. 21, lines 436-437)

“In recent studies, researchers have found that children’s emotion recognition is also affected by facial masks [15, 16, 47].”

Again, thank you for giving us the opportunity to strengthen our manuscript with your valuable comments and queries. We have worked hard to incorporate your feedback and hope that these revisions persuade you to accept our submission.

Sincerely,

Eunsoo Choi

Assistant Professor

School of Psychology

Korea University

Sungbuk-gu, Seoul

South Korea

Email: taysoo@korea.ac.kr

Tel: +82-2-3290-2062
