# Supplementary material for: Impact of face masks and sunglasses on emotion recognition in South Koreans
Source: PLoS One. 2022 Feb 3;17(2):e0263466. doi: 10.1371/journal.pone.0263466 (PMC8812856; doi:10.1371/journal.pone.0263466)
Supplement: Attachment — Submitted filename: Response to Reviewers.docx [file pone.0263466.s002.docx]

**Rebuttal Letter**

Dear Managing Editor,

PLOS ONE Reviews

Thank you for inviting us to submit a revised draft of our manuscript entitled, “Does masking one's facial area masks emotions? The impact of face masks and sunglasses on emotion recognition in South Koreans (revision)” to PLOS ONE. We express our cordial gratitude for the valuable and constructive review of our paper. The reviewers’ comments have helped us to further strengthen the overall quality of the paper. We have incorporated changes that reflect the detailed suggestions you have graciously provided. We also hope that our edits and the responses we provide below satisfactorily address all the issues and concerns you and the reviewer have noted. The specific responses to the reviewer’s comments are listed below.

**The Reviewer's comments:**

**Comment 1:** The effect of gender was not significant as you explained in your letter; please state 1-2 sentences whether this was expected or not and on which basis you argue pro or contra such effects.

**Response:** Although gender was not a major concern of our study, we expected two main effects of gender based on previous studies. First, we expected that women would show higher recognition rates than men. Second, we expected the participants to perform better when the target face is of opposite sex. However, the results showed that the main effect of participant sex and the interaction between participant gender and the gender of target were not significant. Unexpectedly, there was a main effect of stimuli gender was significant. Specifically, we found that participants perceived the emotions of female (vs. male) faces more accurately. As we know of no theoretical reason for this result, more research is needed to replicate such findings first.

The following paragraph is added in the revised manuscript.

“Finally, participant gender did not have any effect on recognition rate nor did they interact with the target’s gender. However, target stimuli’s gender mattered. Overall, participants recognized female faces better than male faces. More research is needed to replicate this effect.” (page 20, lines 410-413)

**Comment 2:** About Comment 4 (from my review before): please use only the precision the base of measurement provides, e.g. age is typically asked regarding years, but not more precise. So any outcome of mean age should not be more precise than about 1/10 of the original precision. So for instance 24.2 instead of 24.23 years.

**Response:** Thank you for your kind explanation. As per your suggestion, we have revised the precision of age by rounding up to the first digit after the decimal point instead of the second digit.

**Comment 3:** [Just a “Thank you!”: Thanks for providing your data and analyses on OSF—the scientific community will appreciate this very much!]

**Response:** Thank you for the reminder. We updated the syntax for the data analyses as well as our figures.

**Comment 4:** Please leave out [Internet] for references

**Response:** We removed [Internet] from the references.

**Comment 5:** IMPORTANT POINT: Fig.3: PLEASE DOUBLE CHECK THIS (just because it is highly unrealistic): was fear really mostly misinterpreted as SURPRISE? I have never seen such a massive systematic confusion of emotional states across different presentation conditions.

**Response:** In this experiment, participants tended to recognize fear as surprise rather than as fear itself. (We were also surprised by these patterns, and we checked the results again several times.) Not only did 41% of participants incorrectly recognize fear as surprise in a mask condition, 65% of participants misread fear as surprise when in uncovered face.

It is true that surprise and fear are often confused (Ekman, 2003) but in our study, the misinterpretation rate was very high. This may suggest that there the facial expression of "fear” in the present facial stimuli set is problematic. In line with this argument, in a study conducted by Kim et al. (2011) where the same KUFEC database was used, participants were not able to make a significant distinction between surprise and fear.

Again, thank you for giving us the opportunity to strengthen our manuscript with your valuable comments and queries. We have worked hard to incorporate your feedback and hope that these revisions persuade you to accept our submission.

Sincerely,

Eunsoo Choi

Assistant Professor

School of Psychology

Korea University

Sungbuk-gu, Seoul

South Korea

Email: taysoo@korea.ac.kr

Tel: +82-2-3290-2062
