# Supplementary material for: Impact of face masks and sunglasses on emotion recognition in South Koreans
Source: PLoS One. 2022 Feb 3;17(2):e0263466. doi: 10.1371/journal.pone.0263466 (PMC8812856; doi:10.1371/journal.pone.0263466)
Supplement: Attachment — Submitted filename: Response to Reviewers.docx [file pone.0263466.s003.docx]

**Rebuttal Letter**

Dear Managing Editor,

PLOS ONE Reviews

Thank you for inviting us to submit a revised draft of our manuscript entitled, “Impact of face masks and sunglasses on emotion recognition in South Koreans (revision)” to PLOS ONE. We express our cordial gratitude for the valuable and constructive review of our paper. The reviewers’ comments have helped us to further strengthen the overall quality of the paper. We have incorporated changes that reflect the detailed suggestions you have graciously provided. We also hope that our edits and the responses we provide below satisfactorily address all the issues and concerns you and the reviewer have noted. The specific responses to the reviewer’s comments are listed below.

**The Editor's comments:**

**Comment 1:** I suggest to shorten the title of your paper to 'Impact of face masks and sunglasses on emotion recognition in South Koreans.

**Response:** As per your suggestion, we have shortened the title to “Impact of face masks and sunglasses on emotion recognition in South Koreans.” We hope that the change will make it easier for readers to access our paper.

**Comment 2:** You also completely ignored my request for more attentive reading of the recent review READING COVERED FACES, and for adding more information on cultural differences on recognition of covered by masks emotions. I hope you can do this promptly.

**Response:** We apologize for carelessly omitting the response to your comment regarding cross-cultural implications of the present research. The current manuscript has added more information on cultural differences on recognition of covered faces by citing studies from your review paper. We greatly appreciate your helpful suggestions.

See p. 5, lines 101-109

" Also, the effects of masks or sunglasses on reading other person's facial expressions may differ depending on the cultural context (for a review see [18]). As an example, consider the findings that a face covered with Islamic headdress such as niqāb impacts the recognition of emotions differently by cultural groups [13, 25], suggesting that a culturally attached meaning of headdress may play a role. As for East Asians, they are not only less accustomed to sunglasses than Westerners, but sunglasses are often considered rude in interpersonal relationships [26, 27]. Given such cultural background, thus, it is necessary to test whether the effects of masks and sunglasses on facial expression recognition that are documented with Western participants would also apply to East Asians.”

Again, thank you for giving us the opportunity to strengthen our manuscript with your valuable comments and queries. We have worked hard to incorporate your feedback and hope that these revisions persuade you to accept our submission.

Sincerely,

Eunsoo Choi

Assistant Professor

School of Psychology

Korea University

Sungbuk-gu, Seoul

South Korea

Email: taysoo@korea.ac.kr

Tel: +82-2-3290-2062
